# Identification and Genomic Characterization of Two Previously Unknown Magnetotactic *Nitrospirae*

**DOI:** 10.3389/fmicb.2021.690052

**Published:** 2021-07-27

**Authors:** Wensi Zhang, Yinzhao Wang, Li Liu, Yongxin Pan, Wei Lin

**Affiliations:** ^1^Key Laboratory of Earth and Planetary Physics, Institute of Geology and Geophysics, Chinese Academy of Sciences, Beijing, China; ^2^France-China Joint Laboratory for Evolution and Development of Magnetotactic Multicellular Organisms, Chinese Academy of Sciences, Beijing, China; ^3^College of Earth and Planetary Sciences, University of Chinese Academy of Sciences, Beijing, China; ^4^State Key Laboratory of Microbial Metabolism, School of Life Sciences and Biotechnology, Shanghai Jiao Tong University, Shanghai, China

**Keywords:** magnetotactic bacteria, *Nitrospirae*, metagenomics, magnetotaxis, magnetosome gene cluster

## Abstract

Magnetotactic bacteria (MTB) are a group of microbes that biomineralize membrane-bound, nanosized magnetite (Fe_3_O_4_), and/or greigite (Fe_3_S_4_) crystals in intracellular magnetic organelle magnetosomes. MTB belonging to the *Nitrospirae* phylum can form up to several hundreds of Fe_3_O_4_ magnetosome crystals and dozens of sulfur globules in a single cell. These MTB are widespread in aquatic environments and sometimes account for a significant proportion of microbial biomass near the oxycline, linking these lineages to the key steps of global iron and sulfur cycling. Despite their ecological and biogeochemical importance, our understanding of the diversity and ecophysiology of magnetotactic *Nitrospirae* is still very limited because this group of MTB remains unculturable. Here, we identify and characterize two previously unknown MTB populations within the *Nitrospirae* phylum through a combination of 16S rRNA gene-based and genome-resolved metagenomic analyses. These two MTB populations represent distinct morphotypes (rod-shaped and coccoid, designated as XYR, and XYC, respectively), and both form more than 100 bullet-shaped magnetosomal crystals per cell. High-quality draft genomes of XYR and XYC have been reconstructed, and they represent a novel species and a novel genus, respectively, according to their average amino-acid identity values with respect to available genomes. Accordingly, the names *Candidatus* Magnetobacterium cryptolimnobacter and *Candidatus* Magnetomicrobium cryptolimnococcus for XYR and XYC, respectively, were proposed. Further comparative genomic analyses of XYR, XYC, and previously reported magnetotactic *Nitrospirae* reveal the general metabolic potential of this MTB group in distinct microenvironments, including CO_2_ fixation, dissimilatory sulfate reduction, sulfide oxidation, nitrogen fixation, or denitrification processes. A remarkably conserved magnetosome gene cluster has been identified across *Nitrospirae* MTB genomes, indicating its putative important adaptive roles in these bacteria. Taken together, the present study provides novel insights into the phylogenomic diversity and ecophysiology of this intriguing, yet poorly understood MTB group.

## Introduction

A diverse group of bacteria in nature can sense and swim along the geomagnetic field, a behavior known as magnetotaxis or microbial magnetoreception (Blakemore, 1975; Lefèvre and Wu, 2013; Lin et al., 2020a). These unique microorganisms, referred to as magnetotactic bacteria (MTB), can actively uptake a large amount of Fe(II) and/or Fe(III) from environments and accumulate it within the cell during the biomineralization of nanosized, membrane-bound Fe_3_O_4_, and/or Fe_3_S_4_ magnetosomes, which are usually arranged in well-ordered, chain-like structures that are responsible for magnetotaxis. Consequently, the intracellular iron content of MTB is much higher (*ca.* 100- to 1,000-fold) than that in other microorganisms (Lin et al., 2014a; Amor et al., 2020a,c). MTB have been discovered across various environments from freshwater to marine ecosystems, and they make important contributions to the global cycling of iron and other elements (such as sulfur, phosphorus, nitrogen, and carbon; Cox et al., 2002; Rivas-Lamelo et al., 2017; Schulz-Vogt et al., 2019; Monteil and Lefèvre, 2020). The determination of the genetic basis for magnetosome biogenesis reveals a complex sequence of steps controlled by dozens of genes clustered at one genomic locus, known as a magnetosome gene cluster (MGC; Grünberg et al., 2001; Murat et al., 2010; Uebe and Schüler, 2016; Lin et al., 2017a; McCausland and Komeili, 2020). For many years, the taxonomic distribution of MTB has been considered to be restricted to a few bacterial phyla (Amann et al., 2006). However, over the past decade, our knowledge of MTB diversity has been significantly expanded through the applications of cultivation-dependent and -independent approaches (Kolinko et al., 2012; Bazylinski et al., 2013; Lefèvre and Bazylinski, 2013; Lin et al., 2017a; Amor et al., 2020b). MTB have thus far been identified in at least 16 phylum-level lineages (Lin et al., 2018, 2020b; Uzun et al., 2020), suggesting unexpected taxonomic diversity of magnetosome biogenesis and magnetotaxis across the domain *Bacteria*.

Magnetotactic bacteria phylogenetically affiliated within the *Nitrospirae* phylum are of great interest because of their biomineralization of up to several hundreds of Fe_3_O_4_ magnetosomal crystals and formation of dozens of sulfur globules in a single cell, linking these microorganisms to the key steps of iron and sulfur cycling in aquatic ecosystems (Spring et al., 1993; Jogler et al., 2010; Lin et al., 2014b; Li et al., 2020). Historically, *Nitrospirae* MTB have only been discovered in freshwater environments (Spring et al., 1993; Flies et al., 2005; Amann et al., 2006); however, recent studies have revealed that these MTB are globally distributed in a wider range of environments than anticipated previously, including hot springs (Lefèvre et al., 2010; Uzun et al., 2020), estuary and marine ecosystems (Lin et al., 2017a; Qian et al., 2019), and acidic peatlands (Lin et al., 2020b). Due to the lack of cultivated representatives, much of our understanding of *Nitrospirae* MTB was obtained through 16S rRNA gene-based methods. Recently, since the first report of a nearly complete draft genome of *Candidatus* (*Ca.*) Magnetobacterium casensis through a targeted metagenomic approach (Lin et al., 2014b), a growing number of *Nitrospirae* MTB genomes have been reconstructed from distinct ecosystems through applications of genome-resolved metagenomics (Lin et al., 2017b, 2018, 2020b; Koziaeva et al., 2020; Uzun et al., 2020) and single-cell genomics (Kolinko et al., 2016). Genomic analyses of *Ca.* Magnetobacterium casensis (Lin et al., 2014b) and *Ca.* Magnetobacterium bavaricum (Kolinko et al., 2016) indicate an autotrophic lifestyle with capacity of CO_2_ fixation via the reductive acetyl-CoA (Wood-Ljungdahl or WL) pathway or reductive TCA (rTCA) pathway. Both species are predicted to conduct denitrification, sulfur oxidation, and/or sulfate reduction in different microenvironments across anoxic and microoxic layers near the oxic-anoxic transition zone (Lin et al., 2014b; Kolinko et al., 2016).

Despite these glimpses into the diversity and metabolic potential of magnetotactic *Nitrospirae*, the vast majority of MTB in this group are underexplored, and a comprehensive phylogenomic and comparative genomic analysis spanning a broad representation of the *Nitrospirae* MTB remains lacking. Here, we report the identification and characterization of two novel *Nitrospirae* MTB populations and the reconstruction of their high-quality draft genome sequences. The newly recovered genomes were further compared with the reported *Nitrospirae* MTB genomes. Findings of this study extend our understanding of the phylogenomic diversity and metabolic potential of this intriguing, yet poorly understood MTB group.

## Materials and Methods

### Observation and Magnetic Enrichment of Magnetotactic Bacteria

Surface sediments (0–10 cm) were collected from freshwater Lake Xianyang (34.33°N 108.72°E) in Xianyang City, Shaanxi Province, China, transferred to 600-ml plastic flasks, covered with approximately 100 ml of lake water and transported to the laboratory. The presence of MTB in sediments was examined using the hanging-drop method (Greenberg et al., 2005) under an Olympus BX51 microscope (Olympus, Tokyo, Japan). MTB cells were magnetically collected using a double-ended open magnetic separation apparatus (referred to as MTB trap) as previously described (Jogler et al., 2009). Briefly, approximately 300 ml of surface sediments were transferred to the MTB trap, and a homogeneous magnetic field was applied for MTB cell enrichment for 4 h. The retrieved MTB cells were then washed with double-distilled water.

### Fluorescence *in situ* Hybridization and Transmission Electron Microscopy

Two distinct morphotypes of MTB have been observed, designated as XYR and XYC. XYR and XYC are abbreviations for Xianyang Lake rods and Xianyang Lake cocci, respectively. 16S rRNA genes were directly amplified from magnetically enriched MTB cells, using universal bacterial primers 27F (5′- AGAGTTTGATCCTGGCTCAG -3′) and 1492R (5′- GGTTACCTTGTTACGACTT -3′). The PCR protocol included denaturation at 94∘C for 3 min; 35 cycles of 94∘C for 45 s, 50∘C for 1 min, and 72∘C for 1.5 min; followed by 10 min at 72∘C and a holding temperature of 4∘C. Sequencing was conducted on 30 randomly selected clones. To identify the newly discovered MTB cells, two oligonucleotide probes specific to 16S rRNA gene sequences of XYR (5′-TCCCTTGCGAGAGTCGTTAT-3′) and XYC (5′- TGCCGAAACACCAGTCGTCC -3′) were designed and applied to *in situ* hybridization of the enriched MTB cells. The candidate probes were manually designed with district regions and further checked using SILVA TestProbe (Quast et al., 2012). The *Ca.* Magnetobacterium bavaricum-specific probe (5′-GCCATCCCCTCGCTTACT-3′, named BaP in this study; Spring et al., 1993) that is specific for most known *Nitrospirae* MTB (Lin et al., 2011) and the 4′,6-diamidino-2′-phenylindole were used as control. Probes for XYR and XYC were synthesized and fluorescently labeled with sulfoindocyanine dye Cy3 at the 5′ ends, and the probe BaP was labeled with fluorescein phosphoramidite FAM at the 5′ end. Fluorescence *in situ* hybridization (FISH) was then performed as previously described (Spring et al., 1993; Pernthaler et al., 2001). Briefly, 2 μl of cell mixtures were dropped onto gelatin-coated glass slides, allowed to dry, and dehydrated through an ethanol gradient (50, 80, and 100% for 3 min each). *In situ* hybridization was performed at 46∘C for 3 h in hybridization buffer with 35% (vol/vol) formamide. A fluorescence microscope Olympus BX51 (Olympus, Tokyo, Japan) was used to record optical sections. For transmission electron microscope (TEM) observation, a 10-μl drop of magnetic enrichment was deposited on a Formvar-carbon-coated copper grid and was allowed to air dry in a laminar flow hood. MTB cells were imaged using a JEM-2100 HR TEM (JEOL, Tokyo, Japan) at 200 kV.

### Metagenomic Sequencing, Assembly, and Genome Binning

Metagenomic sequencing, assembly, and MTB genome binning were performed as previously described (Zhang et al., 2020). Briefly, metagenomic DNA was directly amplified from magnetically enriched cells using the Genomiphi V2 DNA amplification kit (GE Healthcare, United States) according to the manufacturer’s protocol. Shotgun sequencing was performed with an Illumina HiSeq 2500 using the pair-end 150 × 150 library with a 270-bp insert size. The metagenomic data set was assembled *de novo* using metaSPAdes (version 3.13.0; Nurk et al., 2017) with default parameters. Initial assembled scaffolds were binned separately using MetaBAT2 (version 2.12.1; Kang et al., 2019), MaxBin2 (version 2.2.4; Wu et al., 2016), CONCOCT (Alneberg et al., 2014), and MyCC (Lin and Liao, 2016), and the high-scoring, non-redundant set of bins were dereplicated and selected using DASTool (Sieber et al., 2018). The completeness and contamination of the acquired genomes were assessed with CheckM (Parks et al., 2015) using the “lineage_wf” workflow. Statistics for each genome were obtained using QUAST (version 4.2; Gurevich et al., 2013). Genomes were annotated using Prokka (version 1.13.3; Seemann, 2014). Reconstructed genomes were checked manually for the presence of magnetosome genes according to the known magnetosome genes from previously reported *Nitrospirae* MTB, including *Ca.* Magnetobacterium casensis (Lin et al., 2014b), *Ca.* Magnetobacterium bavaricum (Kolinko et al., 2016), and *Ca*. Magnetominusculus xianensis (Lin et al., 2017b). The average amino acid identity (AAI) and average nucleotide identity (ANI) values were calculated using enveomics (Rodriguez-R and Konstantinidis, 2016). The percentage of conserved proteins (POCP) was calculated using BLASTP according to Qin et al. (2014).

### Phylogenetic Analyses

For genome-based phylogenetic analyses, all available magnetotactic *Nitrospirae* genomes (*n* = 38) and non-MTB *Nitrospirae* genomes (a total of 138 GTDB species representatives) as of October 2020 were downloaded from the NCBI (Agarwala et al., 2018) and GTDB (Parks et al., 2018) databases. Those non-MTB genomes were further dereplicated using dRep (Olm et al., 2017) with “-sa 0.99” for dereplication at 99% ANI. The phylogenomic tree was inferred from 120 concatenated bacterial single-copy marker proteins as described previously (Parks et al., 2017) and was calculated using IQ-TREE (version 1.6.9; Nguyen et al., 2015) under the “TEST” option for the best-fit substitution model selection (LG + F + I + G4) with 1,000 ultrafast bootstraps. The genome tree was rooted with *Thermodesulfobacterium geofontis* OPF15 and *Thermodesulfobacterium commune* DSM 2178.

For a 16S rRNA gene-based tree, 16S rRNA gene sequences retrieved from those two genomes reconstructed in this study were searched against NCBI’s nucleotide collection (nr/nt) using BLASTn. The hits with percentage identity ranging from 100 to 90% and query coverage from 100 to 95% were downloaded and aligned by SINA (Pruesse et al., 2012). trimAL (Capella-Gutiérrez et al., 2009; -gappyout) was performed to eliminate poorly aligned portions of the alignment. A phylogenetic tree was subsequently constructed using IQ-TREE (Nguyen et al., 2015) under the TEST option for best model selection (TIM3 + F + I + G4) with 1,000 ultrafast bootstraps. The tree was rooted with *Thermodesulfobacterium geofontis* OPF15 and *Thermodesulfobacterium commune* DSM 2178.

### Comparative Genomic Analysis and Metabolic Predictions

For comparative genomic analysis, only MTB genomes from the *Nitrospirae* phylum with >90% completeness and <5% contamination were considered. These genomes were further dereplicated using dRep (Olm et al., 2017) with “-sa 0.99” for dereplication at 99% ANI. Finally, a total of 13 high-quality representative *Nitrospirae* MTB genomes were selected (including XYR and XYC reconstructed in the present study). These genomes were annotated against the KEGG database using the KofamKOALA server (Aramaki et al., 2020). An overview of the completeness of general metabolic pathways was estimated and visualized using KEGG-Decoder (Graham et al., 2018), and a heat map was generated using “static” visualization mode. Putative magnetosome genes of these genomes were checked using NCBI PSI-BLAST (Altschul et al., 1997) and further manually inspected. Comparison of MGCs were performed with clinker (Gilchrist and Chooi, 2021).

### Data Availability

Genome sequences of *Ca.* Magnetobacterium cryptolimnobacter strain XYR and *Ca.* Magnetomicrobium cryptolimnococcus strain XYC have been deposited in GenBank under the accession numbers JAGYWH000000000 and JAGYWI000000000 (BioProject number PRJNA400260).

## Results and Discussion

### Two Novel Magnetotactic Bacteria Populations Affiliated Within the *Nitrospirae* Phylum

**FIGURE 1:**
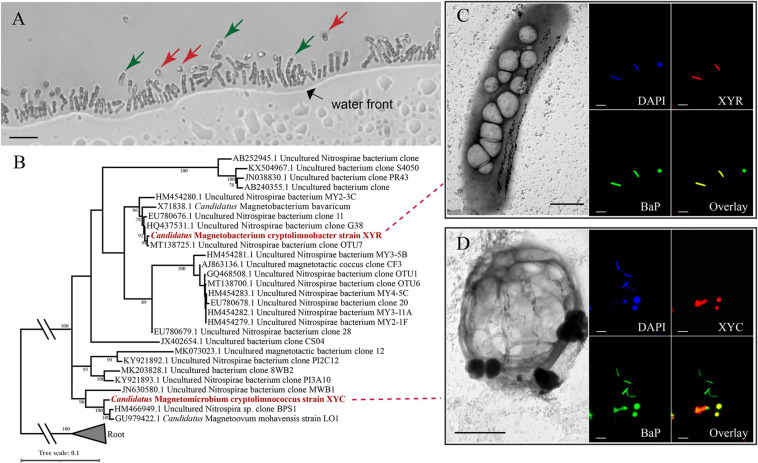
Phylogenetic and morphological identification of two novel *Nitrospirae* MTB. **(A)** Morphologies of *Ca*. Magnetobacterium cryptolimnobacter strain XYR cells (green arrows) and *Ca*. Magnetomicrobium cryptolimnococcus strain XYC cells (red arrows) as revealed by light microscopy (bar = 10 μm). **(B)** Phylogenetic tree based on comparative sequence analysis of 16S rRNA genes. Numbers at nodes are bootstrap support values (*n* = 1,000, only bootstrap values greater than 75% are shown). **(C,D)** Transmission electron microscopy pictures (bar = 1 μm) and fluorescence *in situ* hybridization results (bar = 10 μm) of cells of XYR and XYC, respectively. BaP is the probe specific for most *Nitrospirae* MTB (Spring et al., 1993; Lin et al., 2011).

Light-microscope observation of surface sediments from freshwater Lake Xianyang showed two distinct morphotypes of MTB (rod-shaped and coccoid, Figure 1A). TEM observation reveals that cells of both rods and cocci form intracellular bullet-shaped magnetosome crystals organized into several bundles of chains (Figures 1C,D). Analysis of retrieved 16S rRNA gene sequences revealed only two populations (defined by a 97% identity threshold) in magnetically enriched MTB cells and FISH results turned out that they were either rods or cocci (Figure 1). To reconstruct their draft genomes, cells of MTB in the sediments were magnetically enriched using the “MTB trap,” and their metagenome was sequenced using Illumina techniques. Assembly and binning resulted in two near-complete genome bins (designated XYR and XYC), which consist of 195 and 91 scaffolds with average GC contents of 48.61 and 37.75%, respectively (Table 1). Genomes of XYR and XYC were 4.23 and 3.58 Mbp and were estimated to be 96.97% complete with 2.73% contamination and 99.94% complete with 1.82% contamination, respectively. Each genome contained a complement of 23S, 16S, and 5S rRNA genes and >18 tRNAs, thus exceeding the high-quality level of the minimum information about a metagenome-assembled genome standard (Bowers et al., 2017). 16S rRNA gene identities (91.98%) indicate that XYR and XYC belong to different genus (Yarza et al., 2014). The average AAI value between XYR and XYC was 53.73%, indicating that they represent organisms from two distinct genera according to Konstantinidis et al. (2017). The ANI value between XYR and XYC was too low (only ∼27%) to effectively define their genetic relationship. The pairwise value of the POCP between XYR and XYC was 50.32%, which is near the proposed genus threshold of 50% (Qin et al., 2014). Taking together, XYR and XYC represent two MTB populations from different genus. BLASTn analysis of 16S rRNA gene sequences of XYR and XYC revealed their best hits to an uncultured *Nitrospirae* bacterium clone OTU7 (GenBank accession number , 99.66% identity) and to *Ca.* Magnetoovum mohavensis strain LO-1 (GU979422, 98.60% identity; Lefèvre et al., 2011), respectively.

**TABLE 1:** Genome statistics of *Ca.* Magnetobacterium cryptolimnobacter strain XYR and *Ca.* Magnetomicrobium cryptolimnococcus strain XYC.

**Name**	***Ca.* Magnetobacterium cryptolimnobacter strain XYR**	***Ca.* Magnetomicrobium cryptolimnococcus strain XYC**
No. of scaffolds	195	91
Total length (bp)	4,233,998	3,588,292
Largest scaffold (bp)	210,971	361,266
N50 (bp)	61,028	130,433
No. of genes	3,839	3,216
No. of putative magnetosome genes	25	23
GC content (%)	48.61	37.75
Completeness (%)	96.97	99.94
Contamination (%)	2.73	1.82

According to FISH results, cells of XYR were a rod-shaped morphotype that was similar to previously characterized *Ca.* Magnetobacterium bavaricum (Spring et al., 1993; Jogler et al., 2010) and *Ca.* Magnetobacterium casensis (Lin et al., 2014b), and the morphology of XYC cells represented coccoid and was similar to those of *Ca.* Magnetoovum mohavensis strain LO-1 (Lefèvre et al., 2011) and MWB-1 (Lin et al., 2012). According to TEM observation, cells of XYR are approximately 1–2 μm in width and 5–7 μm in length and contain up to 150 magnetosome crystals organized into two to three bundles of chains. The size of XYC cells ranges from 2 to 4 μm, containing approximately 50–145 magnetosome crystals that formed four to eight bundles of chains. The sizes of magnetosome crystals of XYR and XYC vary from 30 to 130 nm and 45 to 135 nm, respectively.

### Phylogenomic Analysis of Magnetotactic *Nitrospirae*

**FIGURE 2:**
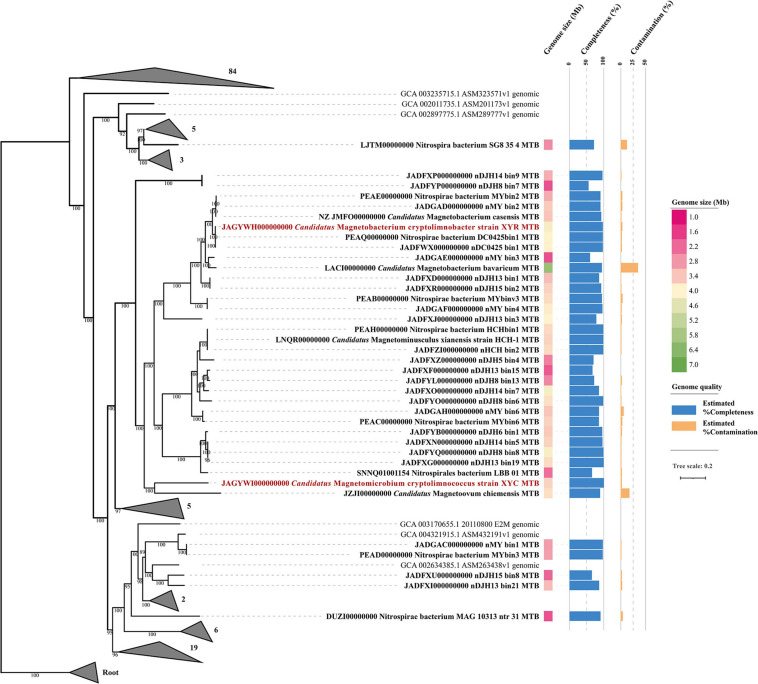
Phylogenomic analysis of 138 *Nitrospirae* genomes including 38 MTB genomes. A maximum likelihood tree was constructed using a concatenated alignment of 120 conserved bacterial markers with GTDB-Tk (120 conserved bacterial marker genes are provided by GTDB). Numbers at nodes are bootstrap support values (*n* = 1,000, only bootstrap values greater than 75% are shown). The genome size and quality of each MTB genome were shown.

Phylogenomic analysis of XYR and XYC and the available *Nitrospirae* MTB genomes was performed based on 120 bacterial single-copy concatenated protein sequence alignment (Figure 2). The genome tree includes 36 previously reported draft genomes of *Nitrospirae* MTB as of October 2020 and 130 representative non-MTB *Nitrospirae* genomes. The majority of *Nitrospirae* MTB genomes are from freshwater lakes (Lin et al., 2014b, 2017b, 2018, 2020b; Kolinko et al., 2016; Koziaeva et al., 2020; Uzun et al., 2020) and acidic peatland soils (Lin et al., 2020b) with one genome (Nitrospirae bacterium SG8_35_4) from a sulfate-rich zone estuary (Lin et al., 2017a) and one (Nitrospirae bacterium MAG_10313_ntr_31) from a hot spring (Uzun et al., 2020). The average estimated completeness and contamination of available *Nitrospirae* MTB genomes was 87.35 ± 12.69% and 3.08 ± 6.15%, respectively. Their genome sizes ranged from 1.91 to 6.08 Mbp (average 3.36 ± 0.82 Mbp) with a genomic GC content from 35.15 to 49.89% (average 45.13 ± 4.19%).

The genome tree placed XYR as a close sister group of *Ca.* Magnetobacterium casensis (Lin et al., 2014b) with an average AAI value of 91.38% and an ANI value of 91.84%, indicating that XYR and *Ca.* Magnetobacterium casensis belong to the same genus but to two different species. Therefore, XYR was provisionally named *Ca.* Magnetobacterium cryptolimnobacter (Ma.gne.to.bac.te′ri.um. Gr. n. magnes, -etos a magnet; N.L. pref. magneto- pertaining to a magnet; N.L. neut. n. bacterium a rod; Cryp.to.lim.no.bac′ter. Gr. adj. kryptos hidden; Gr. fem. n. limne lake; N.L. masc. n. bacter a rod) while XYC represents a separated phylogenetic lineage of the genus level, sharing 55.48% AAI and 50.30% POCP with *Ca.* Magnetoovum chiemensis (Kolinko et al., 2016). XYC was accordingly designated *Ca.* Magnetomicrobium cryptolimnococcus (Ma.gne.to.mi.cro′bi.um. Gr. n. magnes, -etos a magnet; N.L. pref. magneto- pertaining to a magnet; N.L. neut. n. microbium a microbe; Cryp.to.lim.no.coc′cus. Gr. adj. kryptos hidden; Gr. fem. n. limne lake; N.L. masc. n. coccus coccus).

All available *Nitrospirae* MTB genomes were affiliated within the order *Thermodesulfovibrionales* except for *Nitrospirae* bacterium SG8_35_4 (belonging to the order UBA6902) based on phylogenomic analysis and GTDB taxonomy analysis (Figure 2). These genomes were classified into four family-level groups with a majority of genomes (*n* = 32) belonging to the family *Magnetobacteriaceae*. Interestingly, the *Magnetobacteriaceae* family exclusively consists of MTB, including previously well-characterized *Ca.* Magnetobacterium casensis (Lin et al., 2014b) and *Ca.* Magnetobacterium bavaricum (Kolinko et al., 2016). Considering that multiple instances of MGC loss may occur across the bacterial tree of life, the *Magnetobacteriaceae* would be interesting and useful to investigate MTB favorable physiological background and mechanisms to maintain magnetosome formation and magnetotaxis during the evolution. However, those possibilities that *Magnetobacteriaceae* may diverge very recently leading to little opportunity to lose MGCs or that non-MTB members in this family have so far not been sequenced yet cannot be fully ruled out.

### Metabolic Potential of Magnetotactic *Nitrospirae*

**FIGURE 3:**
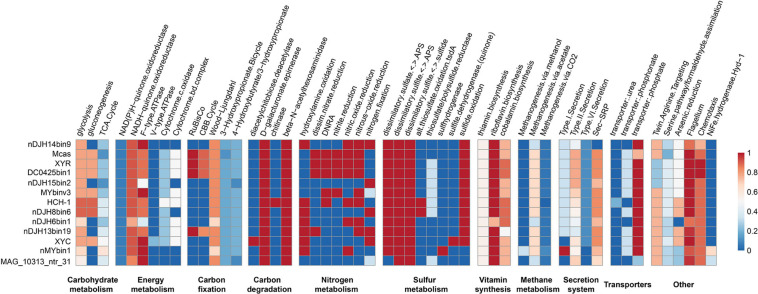
Completeness of metabolic pathways of representative *Nitrospirae* MTB genomes. The scale number represents the completeness of major metabolic pathways inferred from the presence or absence of genes as determined by the KEGG-Decoder. Dark red represents a complete or highly complete pathway, and dark blue represents a pathway that is absent or highly incomplete. nDJH14bin9, *Nitrospirae bacterium* nDJH14bin9; Mcas, *Ca*. Magnetobacterium casensis; HCH-1, *Ca*. Magnetominusculus xianensis strain HCH-1; XYR, *Ca.* Magnetobacterium cryptolimnobacter strain XYR; XYC, *Ca.* Magnetomicrobium cryptolimnococcus strain XYC; DC0425bin1, *Nitrospirae bacterium* DC0425bin1; nDJH15bin2, *Nitrospirae bacterium* nDJH15bin2; MYbinv3, *Nitrospirae bacterium* MYbinv3; nDJH8bin6, *Nitrospirae bacterium* nDJH8bin6; nDJH6bin1, *Nitrospirae bacterium* nDJH6bin1; nDJH13bin19, *Nitrospirae bacterium* nDJH13bin19; nMYbin1, *Nitrospirae bacterium* nMYbin1; and MAG_10313_ntr31, *Nitrospirae bacterium* MAG_10313_ntr31.

Thirteen high-quality, representative *Nitrospirae* MTB genomes were annotated and compared in this study, which revealed that members of *Nitrospirae* MTB generally encoded similar sets of functional genes (Figure 3). Similar to previous studies (Lin et al., 2014b; Kolinko et al., 2016), all these microorganisms encode autotrophic lifestyles with potential capacity in fixation of CO_2_ via the Wood–Ljungdahl pathway. Although RubisCO-encoding genes were identified in four of 13 genomes (including XYR and *Ca.* Magnetobacterium casensis), the lack of carboxylase and oxygenase activity of these genes as previously suggested (Jogler et al., 2010) indicate that *Nitrospirae* MTB might not operate the Calvin–Benson–Bassham (CBB) cycle for CO_2_ fixation.

Genomes of *Nitrospirae* MTB encode proteins involved in the energy-producing dissimilatory sulfate-reduction pathway, suggesting that these microorganisms could reduce sulfate to sulfite and further to sulfide in anoxic microenvironments (Figure 3). In 11 of 13 genomes, we identified the potential for sulfide oxidation to elemental sulfur or sulfite either by sulfide:quinone oxidoreductase (Sqr) or by dissimilatory sulfite reductase complex (Dsr). Sulfite might be further oxidized to sulfate by adenosine 5′-phosphosulfate reductase (Apr) and 5′-triphosphate sulfurylase (Sat). Different from other *Nitrospirae*, the genome of XYC contains a small gene cluster encoding sulfite dehydrogenase (quinone) subunits SoeABC that may catalyze the oxidation of sulfite to sulfate in the cytoplasm (Dahl et al., 2013).

A nearly complete nitrogen fixation pathway has been identified in five *Nitrospirae* MTB genomes (including XYC), which indicates the potential of these MTB to reduce atmospheric molecular nitrogen to ammonia (Figure 3). Several genomes appeared to perform the dissimilatory nitrate reduction to ammonium pathway. Consistent with previous studies (Lin et al., 2014b; Kolinko et al., 2016), the majority of *Nitrospirae* MTB may conduct a denitrification process in which nitrate or nitrite is reduced as a terminal electron acceptor to produce gaseous nitrogen compounds under low-oxygen or anoxic conditions.

These bacteria also encode proteins involved in a series of B-vitamin biosynthesis, including thiamin (B1), riboflavin (B2), and cobalamin (B12). Not surprisingly, genes encoding components of the flagellar and chemotaxis machinery are present in all *Nitrospirae* MTB genomes because magnetotaxis is a flagellum-dependent motility that is generally recognized to be interconnected with chemotaxis (Frankel et al., 1997). Magnetotactic *Nitrospirae* may use the Sec-SRP pathway and the type II secretion system to secrete a variety of proteins and other molecules to the extracellular space. Although a few genes responsible for the type I and VI secretion systems have also been identified in their genomes, whether *Nitrospirae* MTB can perform these pathways needs further experimental confirmation.

### A Remarkably Conserved Magnetosome Gene Cluster Across Magnetotactic *Nitrospirae*

**FIGURE 4:**
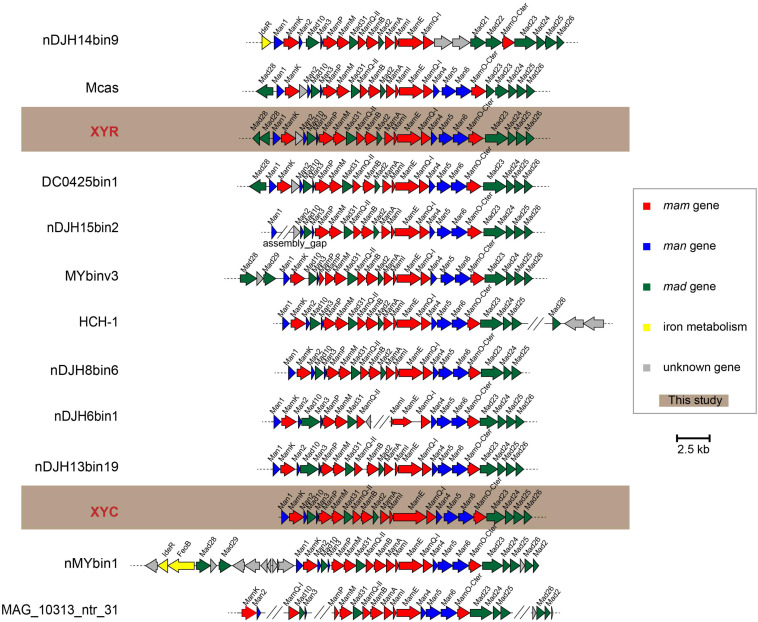
Comparison of MGCs recovered in this study with previously reported representative MGCs. Mcas, *Ca*. Magnetobacterium casensis; HCH-1, *Ca*. Magnetominusculus xianensis strain HCH-1; XYR, *Ca.* Magnetobacterium cryptolimnobacter strain XYR; XYC, *Ca.* Magnetomicrobium cryptolimnococcus strain XYC; DC0425bin1, *Nitrospirae bacterium* DC0425bin1; nDJH15bin2, *Nitrospirae bacterium* nDJH15bin2; MYbinv3, *Nitrospirae bacterium* MYbinv3; nDJH8bin6, *Nitrospirae bacterium* nDJH8bin6; nDJH6bin1, *Nitrospirae bacterium* nDJH6bin1; nDJH13bin19, *Nitrospirae bacterium* nDJH13bin19; nMYbin1, *Nitrospirae bacterium* nMYbin1; and MAG_10313_ntr31, *Nitrospirae bacterium* MAG_10313_ntr31.

Genes responsible for biosynthesis and organization of magnetosomes are clustered in all known MTB genomes, referred to as an MGC (Grünberg et al., 2001; Murat et al., 2010; Lin et al., 2017a). We have identified nearly complete MGCs in both genomes of XYR and XYC (Figure 4). Although XYR and XYC belong to different genera, the gene content and organization of MGCs between them are remarkably conserved, both of which contain genes homologous to magnetosome genes *man1, mamK, man2, mad10, man3, mamP, mamM, mad31, mamQ-II, mamB, mad2, mamA, mamI, mamE, mamQ-I, man4, man5, man6, mamO, mad23, mad24, mad25*, and *mad26.* This Man1-Mad26 gene cluster is across a region of approximately 19 kb in length. Two *mad28* genes located upstream of *man1* of XYR are not identified in the same region of the MGC of XYC (Figure 4).

A further manual check of 13 representative *Nitrospirae* MTB genomes revealed the high stability of the *man1*-*mad26* gene cluster. A total of nine genomes have been identified to contain a complete *man1*-*mad26* gene cluster in a single scaffold except for the lack of the *mad26* gene in the genome of nDJH8bin6 (Figure 4). Both gene content and gene order of *man1*-*mad26* gene clusters are extraordinarily conserved across the *Nitrospirae* MTB from different environmental conditions with the only exception being nDJH14bin9, which suggests that magnetosome biosynthesis and magnetotaxis play important adaptive roles in this MTB group. Alternatively, recent horizontal gene transfer(s) of MGCs into this group or unknown selective pressure(s) to maintain the content and order of magnetosome genes are also possible. A group of six magnetosome genes (*mamABIKMQ*) was identified previously to be shared by the majority of MTB genomes across different bacterial phyla (Lin et al., 2020b). All these six genes have been identified in the *man1*-*mad26* gene cluster. Proteins encoded by these genes are identified to be either essential for magnetosomal biogenesis (*mamBIMQ*) or responsible for important accessory functions, including magnetosome membrane assembly (*mamA*) and magnetosome chain formation (*mamK*) in *Magnetospirillum* strains (Murat et al., 2010; Lohße et al., 2014; Uebe and Schüler, 2016). Similar to their counterparts in *Magnetospirillum*, *Nitrospirae* MamE and MamP contain the PDZ domain that is often involved in organizing signaling complexes by forming protein–protein interactions. MamE is involved in localization of magnetosome proteins and in magnetosomal crystal maturation (Murat et al., 2010; Siponen et al., 2012), and MamP is responsible for redox control of iron biomineralization (Jones et al., 2015) in *Magnetospirillum*. *Nitrospirae* MamO-Cter contains a predicted TauE-like transporter domain and its homolog MamO in *Magnetospirillum*, and homologs MamO and TauE in *Desulfovibrio magneticus* RS-1 has been speculated to be directly involved in nucleation of iron oxide particles (Quinlan et al., 2011; Rahn-Lee et al., 2015).

In addition to the abovementioned Mam proteins with homology to well-characterized magnetosome proteins, the *man1*-*mad26* gene cluster contains additional genes (*man* and *mad* genes) with no apparent homology to MTB belonging to the *Magnetospirillum* (Figure 4). Among these genes, only *mad2* has been functionally characterized previously. Mutant of *mad2* in *Desulfovibrio magneticus* RS-1 resulted in cells containing rare, unusual-looking particles with no magnetic response, indicating the important role of Mad2 in magnetosome biomineralization in this magnetotactic bacterium (Rahn-Lee et al., 2015). Although functions of the other *man* and *mad* genes in the *Nitrospirae* have not been investigated so far, their remarkable conservation suggests that they should govern important functions in magnetosome formation and magnetotaxis in *Nitrospirae* MTB.

Despite the stability of *man1*-*mad26* gene clusters, a few differences were noticed across *Nitrospirae* MTB. For example, genes of *man4*, *man5*, and *man6* are absent from MGC of nDJH14bin9 but are present in those of other magnetotactic *Nitrospirae*, and MGC of nDJH14bin9 contains *mad21* and *mad22* genes that are absent from other *Nitrospirae* MGCs (Figure 4). A gene between *mamK* and *man2* encoding a Uma2 family endonuclease is found in MGCs of XYR, *Ca.* Magnetobacterium casensis and DC0425bin1 but is absent from the rest of the genomes (Figure 4).

## Conclusion

In summary, we have identified and characterized two novel MTB populations, *Ca.* Magnetobacterium cryptolimnobacter strain XYR and *Ca.* Magnetomicrobium cryptolimnococcus strain XYC, belonging to the *Nitrospirae* phylum, which expand the genomic diversity of this MTB lineage. Thoroughly comparative analyses of *Nitrospirae* MTB genomes reveal metabolic plasticity of this widely distributed MTB group and a remarkably conserved MGC. Results of this study deepen our understanding of the taxonomic diversity, genomic property, ecophysiology, and magnetosome biogenesis of MTB members within the *Nitrospirae* phylum. In the future, successful pure cultivation and a combination of metagenomics, metatranscriptomics, and other culture-independent analyses will further deepen our understanding of MTB belonging to the *Nitrospirae* phylum.

## Data Availability Statement

The original contributions presented in the study are publicly available. This data can be found here: Genome sequences of *Ca.* Magnetobacterium cryptolimnobacter strain XYR and *Ca.* Magnetomicrobium cryptolimnococcus strain XYC have been deposited in GenBank under the accession numbers JAGYWH000000000 and  (BioProject number PRJNA400260).

## Author Contributions

WL and WZ conceived and designed the study. YW collected field samples. WZ conducted laboratory work. WZ and WL analyzed the data. WZ and WL wrote the manuscript with input from YW, LL, and YP. All the authors approved the final manuscript.

## Conflict of Interest

The authors declare that the research was conducted in the absence of any commercial or financial relationships that could be construed as a potential conflict of interest.

## Publisher’s Note

All claims expressed in this article are solely those of the authors and do not necessarily represent those of their affiliated organizations, or those of the publisher, the editors and the reviewers. Any product that may be evaluated in this article, or claim that may be made by its manufacturer, is not guaranteed or endorsed by the publisher.
